# Roles of PI3K/AKT/GSK3/mTOR Pathway in Cell Signaling of Mental Illnesses

**DOI:** 10.1155/2012/752563

**Published:** 2012-12-18

**Authors:** Yasuko Kitagishi, Mayumi Kobayashi, Kanae Kikuta, Satoru Matsuda

**Affiliations:** ^1^Department of Environmental Health Science, Nara Women's University, Kita-Uoya Nishimachi, Nara 630-8506, Japan; ^2^Department of Food Science and Nutrition, Nara Women's University, Nara 630-8506, Japan

## Abstract

Several pharmacological agents acting on monoamine neurotransmission are used for the management of mental illnesses. Regulation of PI3K/AKT and GSK3 pathways may constitute an important signaling center in the subcellular integration of the synaptic neurotransmission. The pathways also modulate neuronal cell proliferation, migration, and plasticity. There are evidences to suggest that inflammation of neuron contributes to the pathology of depression. Inflammatory activation of neuron contributes to the loss of glial elements, which are consistent with pathological findings characterizing the depression. A mechanism of anti-inflammatory reactions from antidepressant medications has been found to be associated with an enhancement of heme oxygenase-1 expression. This induction in brain is also important in neuroprotection and neuroplasticity. As enzymes involved in cell survival and neuroplasticity are relevant to neurotrophic factor dysregulation, the PI3K/AKT/GSK3 may provide an important signaling for the neuroprotection in depression. In this paper, we summarize advances on the involvement of the PI3K/AKT/GSK3 pathways in cell signaling of neuronal cells in mental illnesses.

## 1. Introduction

Mental illnesses are a public health concern worldwide [1]. Among them, emotional disorders such as irritability, aggression, and posttraumatic stress disorder are frequently associated with major depression [2]. Although the brain structures responsible for the pathologies are not yet precisely defined, these manifestations are associated with functional alterations of monoamine neurotransmitters expressed by specific neurons [3]. Actually, several pharmacological agents acting on monoamine neurotransmission are used for the management of these disorders. There are similarities between depression and sickness behaviors [4]. Inflammation signaling may provoke the responses with generating neuroprotective effects and neurodegenerative processes [4]. Glycogen synthase kinase 3 (GSK3) may be a sensor determining the neuronal cell fate in the brain [5]. However, there is little understanding of the molecular mechanisms responsible for the therapeutic effects, suggesting that the regulation of the GSK3 is altered in psychiatric disorders [6–8]. In addition, it has been indicated that GSK3 has a role for the regulation of serotonin receptor cell surface trafficking [9]. Several studies even suggest that activation of GSK3 could be an outcome of some susceptibility genes for mental disorders. A similar observation can be made for the potential contribution of AKT to the etiology of mental disorders [10]. Thus, regulation of AKT and GSK3 may constitute an important signaling center in the integration of monoamine neurotransmissions.

**Figure 1:**
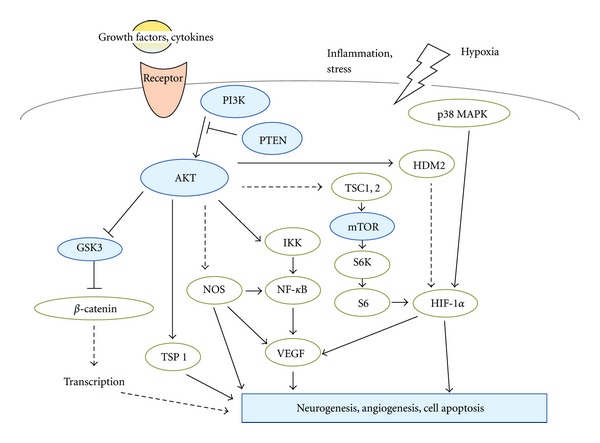
Schematic representation of PI3K/AKT/GSK3/mTOR signaling. Examples of molecules known to act on the regulatory pathways are shown. Note that some critical pathways have been omitted for clarity.

Accumulating evidences suggest that the pathology of depression might be associated with neuronal inflammation [11], which could be attenuated by pharmacological treatment. Because phosphatidylinositol 3-kinase (PI3K) and serine/threonine protein kinase AKT (also known as protein kinase B) seem to make immune cell activation by regulation of the key inflammatory cytokines [12], changes in AKT and GSK3 signaling may contribute to specific therapeutic effects for the depression. Brain intracellular signal transduction systems including the AKT/GSK3 pathway have been found to be altered in patients with psychiatric illnesses [13]. In addition, recent studies have indicated that both dopamine and serotonin exert part of their actions by modulating the activity of AKT/GSK3 [14]. In this paper, we provide an overview of research on the characterization of the regulation of PI3K/AKT/GSK3/mTOR signaling (Figure 1) from the viewpoint of pathogenesis on mental illnesses. Understanding those regulations may provide a better understanding of the major depression, leading to better efficacy of new therapeutic approaches.

## 2. PI3K/AKT Pathway Involved in Major Depression

There are evidences to suggest that inflammation of neuron and inflammatory cytokine production contribute to the pathology of major depression [15–18]. For example, depressed patients have been found to have higher levels of proinflammatory cytokines such as IL-1b, IL-6, TNF-*α*, and IFN-*γ*. Behavioral changes induced by those proinflammatory cytokines in animal model look like symptoms of the depression. Actually, inflammatory cytokines are involved in neurotransmitter metabolisms and synaptic plasticity, and inflammation, which may characterize the depression. The activation of AKT leads to the phosphorylation of GSK3*β*, which is active in resting cells, but is inactivated by the phosphorylation. The GSK3*β* has been linked to the regulation of an assembly of transcription factors, including **β**-catenin, nuclear factor *κ*B (NF-**κ**B), AP-1, NF-AT, and CREB [19]. Thus, the altered activity of GSK3*β* causes various effects on cytokine expression. Activation of PI3K also results in the inhibition of proinflammatory incidents such as expression of IL-12 and TNF-*α*. In addition, the PI3K/AKT/GSK3 pathways (Figure 1) have also emerged as important regulators for type I interferon production. Remarkably, PI3K and mTOR seem to upregulate the anti-inflammatory cytokines and to inhibit the proinflammatory cytokines [20, 21]. 

Neurotransmission mediated by dopamine and serotonin is a major target for psychiatric drugs. Reuptake inhibitors that elevate synaptic serotonin levels are commonly used for the treatment of major depression. Cell-mediated immune activation and inflammation contribute to depressive symptoms, which are in part mediated by increased levels of proinflammatory cytokines such as IL-1, IL-2, IL-6, TNF-*α*, and IFN-*γ* [21]. Fluoxetine is an antidepressant drug which inhibits the reuptake of serotonin in the central nervous system [22]. Studies have shown that fluoxetine can promote neurogenesis and improve the survival rate of neurons [23]. The fluoxetine upregulates expression of the phosphorylated AKT protein, which is related to the neuronal cell survival [23]. Antidepressant drugs have also been shown to promote neuroprotection against neuronal cell death. The mood stabilizer lithium has been known to inhibit GSK3 [24]. In transgenic mice overexpressing tau protein, injection of lithium increases phosphorylation of GSK3*β* with the reduction of GSK3*β* activity in brain, and the axonal degeneration is attenuated in the lithium-treated mice [25]. A mechanism of anti-inflammatory response from antidepressant treatments has been found to be associated with an enhancement of heme oxygenase-1 (HO-1) [26]. The HO-1 is an inducible enzyme that can catalyze the conversion of the heme with potent antioxidant and antiapoptotic activities [27], which are regulated by the PI3K/AKT signaling in response to various inflammatory cytokines. The HO-1 protects against the cytotoxicity of oxidative stress. The induction of HO-1 in brain is important for neuroprotection and neuroplasticity, which are characteristics of antidepressant mechanisms. Guanosine can afford protection against mitochondrial oxidative stress by the PI3K/AKT/GSK3 signaling [28] and by induction of the antioxidant enzyme HO-1 [29]. Melatonin also prevents hemorrhagic shock-induced injury through an AKT-dependent HO-1 expression in animal model [30]. Activation of PI3K/AKT/GSK3 signaling may not only upregulate the HO-1 expression, but also the protective effects of this pathway may be linked to the effects of HO-1. Thus, AKT and GSK3 have been associated with the action of psychiatric drugs.

## 3. Function and Characterization of the PI3K/AKT/mTOR/GSK3 Pathways

The PI3K pathways are known for regulating metabolism, cell growth, and cell survival [31]. The active form of PI3K is an oncogene, and amplifications and mutations of PI3K are commonly found in many kinds of human cancers [31, 32]. The PI3K in mammalian cells forms a family that can be divided into three classes based on the structure, distribution, and mechanism of activation. Class I PI3Ks are divided into class IA and class IB based on different associated adaptors. Class IA PI3Ks are activated by receptor tyrosine kinases, while class IB PI3Ks are activated by G-protein-coupled receptors. These PI3Ks are heterodimers consisting of a regulatory subunit such as p85 and a catalytic subunit such as p110. The PI3K is also known to be required to control cell migration and angiogenesis. The phospholipid second messengers generated by the PI3Ks provide a common mechanism for multiple steps during intracellular signal transduction. 

**Figure 2:**
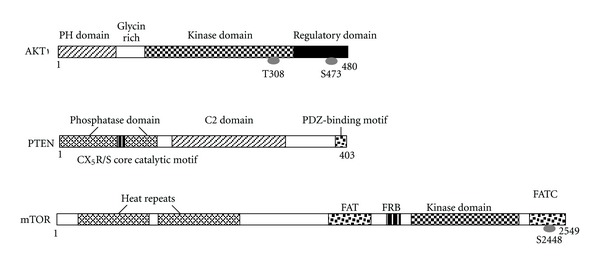
Schematic structures of AKT1, PTEN, and mTOR protein. The predicted consensual domain structures for each protein are depicted. The functionally important sites including the sites of protein phosphorylation are also shown. Note that the sizes of protein are modified for clarity. PH domain: pleckstrin homology domain; C2 domain: a protein structural domain involved in targeting proteins to cell membranes; PDZ: a common structural domain in signaling proteins (PSD95, Dlg, ZO-1, etc.); HEAT: huntington, elongation factor 3, a subunit of PP2A, and TOR1; FAT: FRAP-ATM-TRRAP; FRB: FKBP12-rapamycin binding; FATC: FAT-C-terminal.

The AKT is a major downstream target of the PI3Ks for regulating cell growth and cell migration [33]. Human AKT has three isoforms: AKT1, AKT2, and AKT3. PIP3, a product of PI3K, binds to AKT and leads to the membrane recruitment of the AKT, and it also binds to phosphoinositide-dependent kinase 1 (PDK1) via their pleckstrin homology (PH) domains, then PDK1 phosphorylates AKT in the kinase domain (Thr 308 in AKT1). For the full activation of AKT, the phosphorylation within the carboxyl-terminal regulatory domain (Ser 473 in AKT1) of AKT by PDK2 is required. Schematic structure of the predicted AKT1 protein is shown in Figure 2. Once activated, AKT moves to the cytoplasm and nucleus, where it phosphorylates, activates, or inhibits many downstream targets to regulate various cellular functions (Figure 1). AKT inhibits the GTPase-activating protein (GAP) activity of the tuberous sclerosis complex 1 (TSC1) and TSC2 complex by phosphorylating TSC2 tuberin protein, leading to the accumulation and activation of the mTOR complex (Figure 1). The mTOR mediates the phosphorylation of the ribosomal protein S6 kinases and eukaryotic translation initiation factor 4E-binding protein 1 leading to the release of the translation initiation factor eIF4E. The GSK3 is also a serine/threonine kinase that was initially identified as playing a role in the regulation of glycogen synthesis in response to insulin receptor stimulation. This molecule has been shown to be involved in cellular proliferation, programmed cell death, embryogenesis, and circadian entrainment in addition to the regulation of glycogenesis [34].

PTEN is a dual-specificity phosphatase which has protein phosphatase activity and lipid phosphatase activity that antagonizes PI3K activity [33, 35]. PTEN gene which encodes 403 residue amino acids locates on chromosome 10q23.3. Schematic structure of the predicted PTEN protein is shown in Figure 2. PTEN negatively regulates the activity of PI3K/AKT signaling through converting phosphatidylinositol 3,4,5-triphosphate (PIP3) into phosphatidylinositol 4,5-bisphosphate (PIP2). Because PTEN protein plays an important role in regulating proliferation and migration of many cancer cells, PTEN is considered as a tumor suppressor. PTEN also modulates angiogenesis via downregulating PI3K/AKT pathways. In addition to suppressing the AKT activation, PTEN also controls the activity of Jun N-terminal kinase (JNK). PTEN knockout endothelial cells cause embryonic lethality due to endothelial cell hyper-proliferation and impaired vascular remodeling [36]. PTEN can be upregulated by early growth-regulated transcription factor 1 through direct binding to the PTEN promoter. In addition, peroxisome proliferator-activated receptor **γ**, p53, and activating transcription factor 2 can also transcriptionally upregulate PTEN, while transforming growth factor (TGF)-**β**, NF-**κ**B, and Jun negatively regulate PTEN expression. Interestingly, rosemary extract represses PTEN expression in K562 leukemic culture cells [37]. Some micro-RNAs such as miR-21, miR-19a, and miR-214 inhibit PTEN through targeting the 3′-untranslated region of PTEN, leading to inhibition of PTEN translation [38]. PTEN activity can also be regulated by the posttranslational regulation including phosphorylation, acetylation, and oxidation [37].

## 4. PI3K/AKT/mTOR Pathway Involved in Angiogenesis

Serotonin, a known neurotransmitter, also functions as a factor to promote angiogenesis [39, 40]. The majority of serotonin is stored in platelets, and the platelets aggregation leads to release of serotonin in thrombotic environment. Through G-protein-coupled receptors, serotonin activates the PI3K/AKT/mTOR/p70S6K phosphorylation signaling, and this activation is similar to that seen in VEGF signaling [39]. This provides insight into the overlapping angiogenic signaling pathways stimulated by serotonin in cancer environment. Accordingly, serotonin, PI3K/AKT, hypoxia-inducible factor-1*α* (HIF-1*α*), and VEGF, all play key roles in regulating the angiogenesis. The PI3K/AKT signaling may also regulate angiogenesis by several downstream targets such as NOS and GSK3*β*, which commonly upregulate HIF-1*α* expression inducing VEGF transcriptional activation. Inhibition of GSK3**β** can upregulate HIF-1**α** expression and increases **β**-catenin activity. Hypoxia induces HIF-1**α** production through the increase of its stability and induces VEGF expression in a HIF-1-dependent manner [41]. PI3K/AKT can suppress TSP1, the endogenous antiangiogenic molecule, in endothelial cells. AKT1 knockout mice showed impaired vascular maturation with decreased expression of TSP-1 and TSP-2, while reexpression of TSP-1 and TSP-2 in mice transplanted with wild-type bone marrow is associated with the angiogenesis [42]. Thus, TSP1 inhibits angiogenesis and endothelial cell proliferation and migration. In contrast, TSP1 is an important factor for vascular smooth muscle cell proliferation and migration. The endothelial NOS (eNOS) is critical for VEGF-triggered postnatal angiogenesis. Several protein kinases, such as AKT, AMP-activated protein kinase (AMPK), and protein kinase A (PKA), are known to activate the eNOS [43]. Among them, AKT has emerged as a central regulator for the eNOS activation by growth factors such as VEGF. Inhibition of AKT activity impairs the phosphorylation of HDM2 (human homologue of Murine Double Minute 2), resulting in the destabilization of the HDM2 [44]. It is known that AKT-dependent phosphorylation of HDM2 causes nuclear translocation of the HDM2 followed by HDM2-mediated inactivation of p53. Overexpression of p70S6 K1 in microvascular endothelial cells enhances tumor growth and angiogenesis [45], while HIF-1**α** siRNA significantly inhibits tumor growth and angiogenesis, suggesting that endothelial p70S6 K1 controls tumor angiogenesis through HIF-1**α**. It is plausible that angiogenesis and neurogenesis might share the common pathways including the PI3K/AKT pathways.

## 5. AKT and GSK3 Signaling Modulators Involved in the Actions of Antipsychotics 

**Figure 3:**
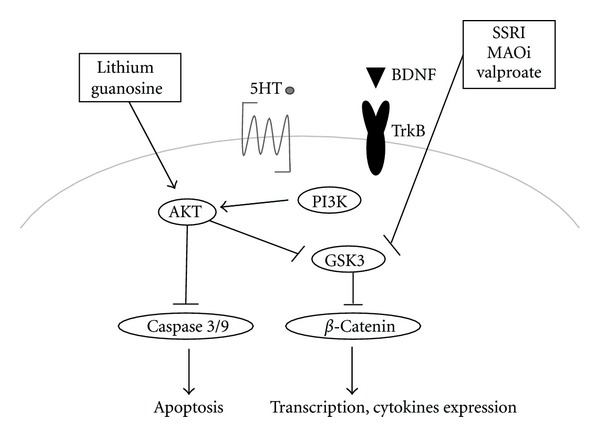
Potential antidepressant molecular targets based on the predominant PI3K/AKT/GSK3 pathways. Note that some critical events have been omitted for clarity. SSRI: selective serotonin reuptake inhibitors; MAOi: monoamine oxidase inhibitors; BDNF: brain-derived neurotrophic factor; 5-HT: 5-hydroxytryptamine, serotonin.

Overall, antidepressants acting on serotonin neurotransmission have been reported to activate AKT and inhibit GSK3 (Figure 3). Several psychoactive drugs have also been shown to modulate the activity of the AKT/GSK3 signaling. AKT has a diverse array of known substrates including the *β*2 subunit of the GABA receptor [46]. Indeed, reductions in AKT activation in neurons may increase excitability through reductions in GABA neurotransmission. Drugs like SSRIs and MAO inhibitors that elevate serotonin synaptic transmission have been shown to inhibit GSK3 (Figure 3). On the contrary, drugs that elevate dopamine neurotransmission reduce the inhibitory phosphorylation of GSK3 and therefore increase the kinase activity. By blocking dopamine D2 receptors, classic antipsychotics can prevent the inhibition of AKT by dopamine and concomitant activation of GSK3. Atypical antipsychotics are also antagonists of serotonin receptors and may interfere with the regulation of GSK3 by the serotonin [47]. Such regulation of AKT and GSK3 activities has also been reported in mice after treatment with haloperidol [48]. Interestingly, AKT/GSK3 pathway is thus regulated by different types of psychiatric drugs, including antidepressants and lithium (Figure 3). Lithium activates PI3K itself, which in turn results in PI3K-dependent phosphorylation and activation of the AKT, then phosphorylation and inactivation of the GSK3, protecting against neuronal toxicity. Therefore, treatment with LY294002 or wortmannin, an inhibitor of PI3K, abolished constitutive activity of AKT, which is attenuated by pretreatment with lithium, then inducing neuronal death. In addition, glutamate-induced reduction of AKT activity as well as the associated neuronal toxicity and caspase-3 activation in apoptosis pathways are prevented by the lithium treatment [48]. Furthermore, several proteins encoded by genes associated with mental disorders affect the activity of this signaling pathway (Figure 3). The mood stabilizers such as valproate have also been reported to inhibit GSK3 [49]. In addition, direct inhibition of GSK3 isoforms has been shown to have effects that are similar to some of those of antidepressants in animal models [50]. Activation of AKT and inhibition of GSK3 may be characterized as fundamental effects for some shared action of psychoactive drugs. 

Guanosine increases AKT and GSK3*β* phosphorylation confirming that this pathway plays an important role in the neuroprotective effect [51]. The guanosine also induces the antioxidant enzyme HO-1 expression. The protective effects of guanosine are partially prevented by HO-1 inhibitor, SnPP. In addition, bilirubin, an antioxidant and physiologic product of HO-1, is protective against oxidative stress. When blocking the AKT pathway with LY294002, a selective inhibitor of PI3K, the neuroprotective effect of guanosine is abolished. Zinc protoporphyrin IX (ZnPP), a selective inhibitor of HO-1, attenuates apoptosis and oxidative stress in PC12 neuronal cells [52]. As H_2_O_2_ preconditioning enhances phosphorylation of AKT, treatment with the LY294002 before H_2_O_2_ preconditioning blocks not only H_2_O_2_-induced HO-1 induction, but also the protective effect of H_2_O_2_ preconditioning against the cytotoxicity. In this way, increasing evidences pointing to AKT pathway modification in depression provide a novel implication of antidepressant mechanisms.

At present, the inhibitors for PI3K/AKT signaling are as follows. Pan-PI3K inhibitors, wortmannin and LY294002, are commonly used to inhibit cancer cell proliferation. Wortmannin is a fungal product, which exerts its effect by the covalent interaction to the conserved Lys802 of the p110**α** catalytic subunit. Both wortmannin and LY294002 crossreact with PI3K-related kinases such as mTOR. A p110**δ** specific inhibitor (IC486068) enhances tumor vascular destruction. The first developed group of AKT inhibitors was lipid-based inhibitors that include perifosine, phosphatidylinositol ether lipid analogs. The perifosine inhibits the translocation of AKT to the cell membrane. Inositol pentakisphosphate, one of the PI3K/AKT inhibitors, also inhibits tumor growth and angiogenesis. Several other AKT antagonists such as 9-methoxy-2-methylellipticinium acetate, indazole-pyridine A-443654, and isoform-specific canthine alkaloid analogs have also been identified and shown to inhibit cancer cell growth and induce apoptosis [53]. The mTOR inhibitors such as rapamycin and its analogs inhibit mTOR activation by binding to FK506-binding protein 12 [54]. Rapamycin and its analogs can activate upstream molecules including AKT [55]. It is important to exploit the potential benefits of the therapies for depression and optimal treatment and/or combination with these inhibitors.

## 6. Perspective

Depression is a condition for which the precise causal factors remain unknown. As antidepressants are expected to be beneficial in the treatment of acute neuronal injuries [56] and chronic progressive neurodegenerative diseases [57], major depression may be a novel class of neurodegenerative disease. It will be valuable to determine proof of the concept in animal models with depressive-like behaviors, for which administration of the new factor induces antidepressant-like effects. However, the use of animal models for studying the major depression has a lot of difficulties, including the lack of a real animal model of the depression. It will be of interest to see whether new treatments alter disease signature genes in neuron cultures or model animal brain. Major depression may be attributed to the impairments of cell survival and cell death signaling pathways in neurons [58], which appear as abnormalities in the functional and morphological plasticity. Increased neuronal cell death and decreased neurogenesis are actually associated with depression, which are prevented or restored by antidepressants. So, antidepressants such as lithium may protect the functional and morphological plasticity in neurons. New drugs with potent strong neuroprotective activity should be developed for the treatment of psychiatric disorders including major depression.

The possible involvement of the PI3K/AKT/GSK3/mTOR in signaling evoked by the neuromonoamine has remained unexplored. Between angiogenesis and neurogenesis, there might be common pathways including the PI3K/AKT/GSK3/mTOR pathway. Whereas many questions remain to be answered about the role of the PI3K/AKT/GSK3/mTOR signaling in mental disorders, it is possible that the inhibition of the signaling in specific neuronal populations could be associated with distinct behavioral outcomes [4, 6, 59]. More understanding of the precise intracellular mechanisms downstream of PI3K/AKT/GSK3/mTOR changes in mental illnesses could provide novel insights into the development of new therapeutic approaches having greater efficacy against major depression.

## Abbreviations

AMPK: AMP-activated protein kinase
BDNF: Brain-derived neurotrophic factor
GAP: GTPase-activating protein
HDM2: Human homologue of Murine Double Minute2
HIF-1*α*: Hypoxia-inducible factor-1*α*
HO-1: Heme oxygenase-1
IL-1b: Interleukin-1beta
IL-2: Interleukin-2
IL-6: Interleukin-6
IFN-*γ*: Interferon-gamma
JNK: Jun N-terminal kinase
mTOR: Mammalian target of rapamycin
NF-*κ*B: Nuclear factor *κ*B
PDK1: Phosphoinositide-dependent kinase 1
PH: Pleckstrin homology
PI3K: Phosphatidylinositol 3-kinase
PKA: Protein kinase A
TNF-*α*: Tumor necrosis factor-alpha
TSC1: Tuberous sclerosis complex 1.
